# 2,4,6-Trimethyl­anilinium chloro­acetate

**DOI:** 10.1107/S1600536811026936

**Published:** 2011-07-09

**Authors:** Rong Tao

**Affiliations:** a Ordered Matter Science Research Center, Southeast University, Nanjing 210096, People’s Republic of China

## Abstract

In the crystal structure of the title compound, C_9_H_14_N^+^·C_2_H_2_ClO_2_^−^, inter­molecular N—H⋯O inter­actions link the mol­ecules into a one-dimensional linear structure.

## Related literature

The title compound was studied as part of our work to obtain potential ferroelectric phase-transition materials. For general background to ferroelectric organic frameworks, see: Ye *et al.* (2006 ▶, 2009 ▶); Fu *et al.* (2007 ▶); for phase transition of ferroelectric materials, see: Zhang *et al.* (2008 ▶); Zhao *et al.* (2008 ▶).
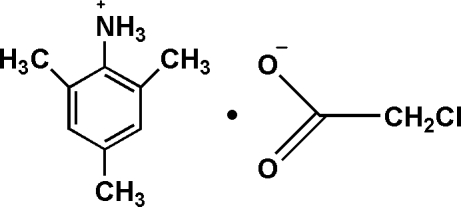


## Experimental

### 

#### Crystal data

C_9_H_14_N^+^·C_2_H_2_ClO_2_^−^
*M* *_r_* = 229.70
Monoclinic, 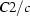

*a* = 26.529 (5) Å
*b* = 4.7453 (9) Å
*c* = 22.717 (5) Å
β = 124.24 (3)°
*V* = 2364.2 (8) Å^3^
*Z* = 8
Mo *K*α radiation
μ = 0.30 mm^−1^
*T* = 293 K
0.20 × 0.20 × 0.20 mm

#### Data collection

Rigaku SCXmini diffractometer
Absorption correction: multi-scan (*CrystalClear*; Rigaku, 2005 ▶) *T*_min_ = 0.941, *T*_max_ = 0.941
11449 measured reflections
2690 independent reflections
1900 reflections with *I* > 2σ(*I*)
*R*_int_ = 0.050

#### Refinement

*R*[*F* ^2^ > 2σ(*F* ^2^)] = 0.057
*wR*(*F* ^2^) = 0.179
*S* = 1.07
2690 reflections
140 parameters
H-atom parameters constrained
Δρ_max_ = 0.30 e Å^−3^
Δρ_min_ = −0.26 e Å^−3^

### 

Data collection: *CrystalClear* (Rigaku, 2005 ▶); cell refinement: *CrystalClear*; data reduction: *CrystalClear*; program(s) used to solve structure: *SHELXS97* (Sheldrick, 2008 ▶); program(s) used to refine structure: *SHELXL97* (Sheldrick, 2008 ▶); molecular graphics: *DIAMOND* (Brandenburg & Putz, 2005 ▶); software used to prepare material for publication: *SHELXL97*.

## Supplementary Material

Crystal structure: contains datablock(s) I, global. DOI: 10.1107/S1600536811026936/jh2308sup1.cif

Structure factors: contains datablock(s) I. DOI: 10.1107/S1600536811026936/jh2308Isup2.hkl

Supplementary material file. DOI: 10.1107/S1600536811026936/jh2308Isup3.cml

Additional supplementary materials:  crystallographic information; 3D view; checkCIF report

## Figures and Tables

**Table 1:** Hydrogen-bond geometry (Å, °)

*D*—H⋯*A*	*D*—H	H⋯*A*	*D*⋯*A*	*D*—H⋯*A*
N1—H1*B*⋯O2	0.89	2.02	2.860 (2)	156
N1—H1*A*⋯O2^i^	0.89	1.88	2.748 (2)	165
N1—H1*C*⋯O1^ii^	0.89	1.93	2.809 (2)	169

Symmetry codes: (i) 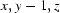; (ii) 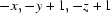.

**Fig. 1.**
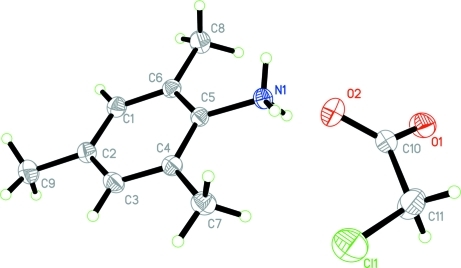
The molecular structure of the title compound, with the atomic numbering scheme.Displacement ellipsoids are drawn at the 30%

**Fig. 2.**
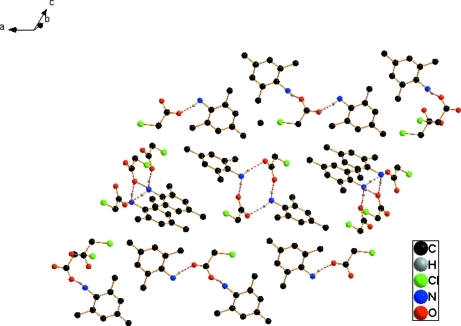
A view of the packing of the title compound, stacking along the b axis. Dashed lines indicate hydrogen bonds.

## supplementary crystallographic information

### Comment

In the crystal structure, one hydrogen-bonding network of N—H···O hydrogen bonds which established between ammonium groups and chloroacetateions, and one kind of intramolecular hydrogen bond which established between N1 and O2 (N1—H···O22.860 (2) Å) contribute to the stability of crystal packing.

In the structure, atom N1 is hydrogen bonded to there O atoms of chloroacetate ions through the normal hydrogen bonds that contain two kind of intermolecular hydrogen bond (N1—H···O2 2.860 (2)Å and N1—H···O1 2.809 (2) Å) and one kind of intramolecular hydrogen bond.

The study of ferroelectric materials has received much attention. Some materials have predominantly dielectric-ferroelectric performance. The title compound was studied as part of our work to obtain potential ferroelectric phase-transition materials (Ye *et al.*, 2006; Fu *et al.*, 2007; Zhao *et al.* 2008; Zhang *et al.*, 2008; Ye *et al.*, 2009). Unluckily, the compound has no dielectric anomalies in the temperature range 93–453 K, suggesting that it might be only a paraelectric.

### Experimental

For the preparation of the title compound, the chloroacetic acid(0.5 g) was added to the ethanol solution of the 2,4,6-trimethylaniline, The resulting precipitate was filtered. Colorless crystals suitable for X-ray analysis were formed after several weeks by slow evaporation of the solvent at room temperature.

### Refinement

Positional parameters of all the H atoms bonded to C atoms were calculated geometrically and were allowed to ride on the C atoms to which they are bonded, with *U*_iso_(H) = 1.2Ueq(C) and *U*_iso_(H) = 1.5Ueq(C) for the methyl group. The other H bonded to N atoms were calculated geometrically and were allowed to ride on the N atoms with *U*_iso_(H) = 1.2Ueq(N).

### Crystal data

**Table:**

C_9_H_14_N^+^·C_2_H_2_ClO_2_^−^	*F*(000) = 976
*M**_r_* = 229.70	*D*_x_ = 1.291 Mg m^−^^3^
Monoclinic, *C*2/*c*	Mo *K*α radiation, λ = 0.71073 Å
Hall symbol: -C 2yc	Cell parameters from 2690 reflections
*a* = 26.529 (5) Å	θ = 3.1–27.5°
*b* = 4.7453 (9) Å	µ = 0.30 mm^−^^1^
*c* = 22.717 (5) Å	*T* = 293 K
β = 124.24 (3)°	Prism, colourless
*V* = 2364.2 (8) Å^3^	0.20 × 0.20 × 0.20 mm
*Z* = 8	

### Data collection

**Table:**

Rigaku SCXmini diffractometer	2690 independent reflections
Radiation source: fine-focus sealed tube	1900 reflections with *I* > 2σ(*I*)
graphite	*R*_int_ = 0.050
Detector resolution: 13.6612 pixels mm^-1^	θ_max_ = 27.5°, θ_min_ = 3.1°
CCD_Profile_fitting scans	*h* = −34→34
Absorption correction: multi-scan (*CrystalClear*; Rigaku, 2005)	*k* = −6→5
*T*_min_ = 0.941, *T*_max_ = 0.941	*l* = −29→29
11449 measured reflections	

### Refinement

**Table:**

Refinement on *F*^2^	Primary atom site location: structure-invariant direct methods
Least-squares matrix: full	Secondary atom site location: difference Fourier map
*R*[*F*^2^ > 2σ(*F*^2^)] = 0.057	Hydrogen site location: inferred from neighbouring sites
*wR*(*F*^2^) = 0.179	H-atom parameters constrained
*S* = 1.07	*w* = 1/[σ^2^(*F*_o_^2^) + (0.0913*P*)^2^ + 1.3591*P*] where *P* = (*F*_o_^2^ + 2*F*_c_^2^)/3
2690 reflections	(Δ/σ)_max_ < 0.001
140 parameters	Δρ_max_ = 0.30 e Å^−^^3^
0 restraints	Δρ_min_ = −0.26 e Å^−^^3^

### Special details

**Table:**

Geometry. All e.s.d.'s (except the e.s.d. in the dihedral angle between two l.s. planes) are estimated using the full covariance matrix. The cell e.s.d.'s are taken into account individually in the estimation of e.s.d.'s in distances, angles and torsion angles; correlations between e.s.d.'s in cell parameters are only used when they are defined by crystal symmetry. An approximate (isotropic) treatment of cell e.s.d.'s is used for estimating e.s.d.'s involving l.s. planes.
Refinement. Refinement of *F*^2^ against ALL reflections. The weighted *R*-factor *wR* and goodness of fit *S* are based on *F*^2^, conventional *R*-factors *R* are based on *F*, with *F* set to zero for negative *F*^2^. The threshold expression of *F*^2^ > σ(*F*^2^) is used only for calculating *R*-factors(gt) *etc*. and is not relevant to the choice of reflections for refinement. *R*-factors based on *F*^2^ are statistically about twice as large as those based on *F*, and *R*- factors based on ALL data will be even larger.

### Fractional atomic coordinates and isotropic or equivalent isotropic displacement parameters (Å^2^)

**Table:**

	*x*	*y*	*z*	*U*_iso_*/*U*_eq_	
C1	0.24425 (10)	−0.0238 (5)	0.67051 (12)	0.0389 (6)	
H1	0.2600	−0.1541	0.7074	0.047*	
C2	0.28257 (11)	0.0902 (5)	0.65328 (12)	0.0396 (6)	
C3	0.25819 (11)	0.2862 (5)	0.59771 (13)	0.0383 (5)	
H3	0.2835	0.3641	0.5858	0.046*	
C4	0.19732 (10)	0.3681 (5)	0.55975 (12)	0.0331 (5)	
C5	0.16059 (9)	0.2490 (4)	0.57926 (11)	0.0298 (5)	
C6	0.18307 (10)	0.0510 (5)	0.63429 (11)	0.0327 (5)	
C7	0.17409 (12)	0.5789 (5)	0.49986 (13)	0.0431 (6)	
H7A	0.1644	0.7526	0.5130	0.065*	
H7B	0.1382	0.5055	0.4576	0.065*	
H7C	0.2050	0.6122	0.4910	0.065*	
C8	0.14313 (11)	−0.0861 (6)	0.65436 (14)	0.0449 (6)	
H8A	0.1633	−0.2502	0.6829	0.067*	
H8B	0.1050	−0.1400	0.6119	0.067*	
H8C	0.1359	0.0450	0.6810	0.067*	
C9	0.34835 (12)	−0.0022 (7)	0.69235 (15)	0.0559 (7)	
H9A	0.3648	−0.0243	0.7420	0.084*	
H9B	0.3714	0.1375	0.6866	0.084*	
H9C	0.3506	−0.1786	0.6733	0.084*	
C10	−0.00154 (10)	0.7701 (5)	0.40030 (12)	0.0354 (5)	
C11	−0.00622 (14)	0.9110 (8)	0.33770 (15)	0.0690 (10)	
H11A	−0.0403	1.0412	0.3163	0.083*	
H11B	−0.0156	0.7677	0.3025	0.083*	
Cl1	0.05862 (4)	1.0959 (2)	0.35657 (5)	0.0832 (4)	
N1	0.09497 (8)	0.3181 (4)	0.53843 (9)	0.0328 (4)	
H1A	0.0737	0.1818	0.5070	0.049*	
H1B	0.0884	0.4804	0.5155	0.049*	
H1C	0.0833	0.3337	0.5681	0.049*	
O1	−0.04344 (8)	0.6016 (4)	0.38346 (10)	0.0499 (5)	
O2	0.04153 (8)	0.8320 (3)	0.46255 (8)	0.0432 (4)	

### Atomic displacement parameters (Å^2^)

**Table:**

	*U*^11^	*U*^22^	*U*^33^	*U*^12^	*U*^13^	*U*^23^
C1	0.0366 (13)	0.0421 (13)	0.0342 (12)	0.0037 (10)	0.0175 (11)	0.0028 (10)
C2	0.0326 (12)	0.0467 (14)	0.0370 (12)	0.0019 (10)	0.0181 (11)	−0.0029 (10)
C3	0.0349 (12)	0.0410 (13)	0.0456 (13)	−0.0045 (10)	0.0267 (11)	−0.0033 (10)
C4	0.0356 (12)	0.0296 (11)	0.0353 (11)	−0.0024 (9)	0.0207 (10)	−0.0051 (9)
C5	0.0284 (11)	0.0288 (11)	0.0305 (11)	−0.0029 (8)	0.0157 (9)	−0.0051 (8)
C6	0.0342 (12)	0.0333 (12)	0.0302 (11)	−0.0033 (9)	0.0180 (10)	−0.0029 (9)
C7	0.0471 (14)	0.0406 (14)	0.0472 (14)	−0.0001 (11)	0.0300 (13)	0.0063 (11)
C8	0.0419 (14)	0.0473 (15)	0.0459 (14)	0.0015 (11)	0.0251 (12)	0.0124 (11)
C9	0.0353 (14)	0.078 (2)	0.0488 (16)	0.0087 (13)	0.0205 (13)	0.0064 (14)
C10	0.0319 (12)	0.0367 (13)	0.0380 (12)	0.0007 (9)	0.0198 (11)	0.0001 (10)
C11	0.0509 (17)	0.103 (3)	0.0427 (16)	−0.0298 (17)	0.0204 (14)	0.0072 (15)
Cl1	0.0700 (6)	0.1098 (8)	0.0800 (6)	−0.0347 (5)	0.0484 (5)	0.0061 (5)
N1	0.0304 (10)	0.0319 (10)	0.0351 (10)	−0.0026 (8)	0.0179 (8)	−0.0005 (8)
O1	0.0449 (10)	0.0583 (12)	0.0523 (11)	−0.0175 (9)	0.0308 (9)	−0.0082 (9)
O2	0.0433 (10)	0.0368 (9)	0.0354 (9)	−0.0011 (7)	0.0136 (8)	0.0001 (7)

### Geometric parameters (Å, °)

**Table:**

C1—C2	1.388 (3)	C8—H8A	0.9600
C1—C6	1.391 (3)	C8—H8B	0.9600
C1—H1	0.9300	C8—H8C	0.9600
C2—C3	1.399 (3)	C9—H9A	0.9600
C2—C9	1.511 (3)	C9—H9B	0.9600
C3—C4	1.391 (3)	C9—H9C	0.9600
C3—H3	0.9300	C10—O1	1.242 (3)
C4—C5	1.397 (3)	C10—O2	1.254 (3)
C4—C7	1.512 (3)	C10—C11	1.512 (4)
C5—C6	1.401 (3)	C11—Cl1	1.756 (3)
C5—N1	1.477 (3)	C11—H11A	0.9700
C6—C8	1.516 (3)	C11—H11B	0.9700
C7—H7A	0.9600	N1—H1A	0.8900
C7—H7B	0.9600	N1—H1B	0.8900
C7—H7C	0.9600	N1—H1C	0.8900
			
C2—C1—C6	122.0 (2)	H8A—C8—H8B	109.5
C2—C1—H1	119.0	C6—C8—H8C	109.5
C6—C1—H1	119.0	H8A—C8—H8C	109.5
C1—C2—C3	118.2 (2)	H8B—C8—H8C	109.5
C1—C2—C9	120.5 (2)	C2—C9—H9A	109.5
C3—C2—C9	121.2 (2)	C2—C9—H9B	109.5
C4—C3—C2	122.1 (2)	H9A—C9—H9B	109.5
C4—C3—H3	119.0	C2—C9—H9C	109.5
C2—C3—H3	119.0	H9A—C9—H9C	109.5
C3—C4—C5	117.6 (2)	H9B—C9—H9C	109.5
C3—C4—C7	119.1 (2)	O1—C10—O2	126.0 (2)
C5—C4—C7	123.3 (2)	O1—C10—C11	114.2 (2)
C4—C5—C6	122.1 (2)	O2—C10—C11	119.8 (2)
C4—C5—N1	119.81 (19)	C10—C11—Cl1	116.1 (2)
C6—C5—N1	117.96 (18)	C10—C11—H11A	108.3
C1—C6—C5	117.9 (2)	Cl1—C11—H11A	108.3
C1—C6—C8	119.5 (2)	C10—C11—H11B	108.3
C5—C6—C8	122.5 (2)	Cl1—C11—H11B	108.3
C4—C7—H7A	109.5	H11A—C11—H11B	107.4
C4—C7—H7B	109.5	C5—N1—H1A	109.5
H7A—C7—H7B	109.5	C5—N1—H1B	109.5
C4—C7—H7C	109.5	H1A—N1—H1B	109.5
H7A—C7—H7C	109.5	C5—N1—H1C	109.5
H7B—C7—H7C	109.5	H1A—N1—H1C	109.5
C6—C8—H8A	109.5	H1B—N1—H1C	109.5
C6—C8—H8B	109.5		

### Hydrogen-bond geometry (Å, °)

**Table:**

*D*—H···*A*	*D*—H	H···*A*	*D*···*A*	*D*—H···*A*
N1—H1B···O2	0.89	2.02	2.860 (2)	156
N1—H1A···O2^i^	0.89	1.88	2.748 (2)	165
N1—H1C···O1^ii^	0.89	1.93	2.809 (2)	169

Symmetry codes: (i) *x*, *y*−1, *z*; (ii) −*x*, −*y*+1, −*z*+1.
